# Comparison of Models for the Collinearity of Hox Genes in the Developmental Axes of Vertebrates

**DOI:** 10.2174/138920212800543093

**Published:** 2012-05

**Authors:** Spyros Papageorgiou

**Affiliations:** Institute of Biology, NCSR ‘Demokritos’, Athens, Greece

**Keywords:** Chromatin, collinearity, Hox genes, limb, mouse, trunk, vertebrate.

## Abstract

Hox gene clusters are very frequent in many animal genomes and their role in development is pivotal. Particularly in vertebrates, intensive efforts have established several properties of Hox clusters. The collinearity of Hox gene expressions (spatial, temporal and quantitative) is a common feature of the vertebrates. During the last decade, genetic engineering experiments have revealed some important facets of collinearity during limb and trunk development in mice. Two models have been proposed to explain all these properties. On one hand the ‘two-phases model’ makes use of the molecular regulatory mechanisms acting on the Hox genes. On the other hand, the’biophysical model’ is based on the signals transduced inside the cell nucleus and the generation of forces which apply on the cluster and lead to a coordinated activation of Hox genes. The two models differ fundamentally and a critical and detailed comparison is presented. Furthermore, experiments are proposed for which the two models provide divergent predictions. The outcome of these experiments will help to decide which of the two models is valid (if any).

## INTRODUCTION

Hox genes are a distinct branch of the homeobox gene superfamily and they are responsible for pattern formation on the head to tail axis of animal embryos [1]. Their location on the chromosome takes divergent forms: they are disorganized and with long intergenic regions as in the sea urchin or they are well organized and compact as in the vertebtrates. The evolutionary interrelation between the two forms has been explicitly studied [2]. (See also the Discussion). 

E.B.Lewis was the first traditional geneticist who noticed the following unexpected feature of collinearity [3]: Hox genes are located in order (Hox1, Hox2, Hox3, …) along the 3’ to 5’ direction on the chromosome. The expressions of these genes follow the same order along the anterior-posterior axis of the* Drosophila *embryo (spatial collinearity). This strange property of Hox gene expressions indicates that a profound correlation is at work between the macroscopic scale of the embryo (of the order of 1 mm) and the microscopic scale of the chromatin domain (of about 500 nm). Typically the scale difference extends in 3 orders of magnitude. This multiscale correlation is an organization characteristic of systems biology [4]. 

**Fig. (1). Schematic representation of the Hox gene expressions:**
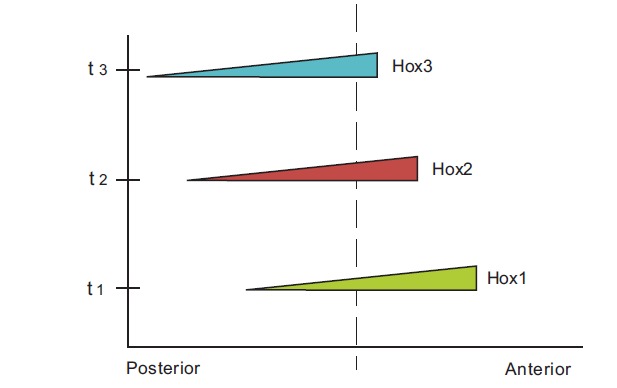
The anterior boundary of Hox1 expression is more anterior than the anterior boundary of Hox2 expression etc. (spatial collinearity). The starting time (t1) of Hox1 expression is earlier than starting time (t2) of Hox2 expression etc (temporal collinearity). At a given position along the anterior-posterior axis (dashed line) the expression of Hox3 is stronger than the expression of Hox2 and Hox1 (quantitative collinearity).

Besides spatial collinearity, it was found that Hox1, Hox2, Hox3,…of a vertebrate Hox cluster are activated sequentially in time following the same order (Fig. **1**): Hox1 is expressed first, later follows the expression of Hox2 etc (temporal collinearity) [5]. Furthermore, a third kind of collinearity was established (Fig. **1**): when at a given position on the anterior-posterior axis several Hox genes are co-activated, the expression of the most posterior gene in the cluster is stronger than the other gene expressions (quantitative collinearity) [6]. 

The origin and establishment of collinearity is not clear. According to Gehring *et al. *[7] collinearity evolved from repeated tandem duplications of an ancestral ur-Hox gene which constitutes the ground state. The duplicates were sequentially modified by evolution in both the anterior and posterior directions. 

Another hypothesis for the origin of collinearity was recently proposed by Durston *et al. *[8]. This model is based on the observed temporal collinearity of the first Hox gene expressions during gastrulation. There is evidence that transacting factors and intercellular interactions may cause Hox collinearity. These interactions include posterior prevalence which is a genetic property according to which posterior Hox genes are dominant compared to anterior Hox genes. Evidence from evolutionary studies establishes posterior prevalence in both *Drosophila* and vertebrates [8]. It is clear that posterior prevalence is related to quantitative collinearity as presented above. 

There is also a mechanistic explanation of collinearity according to which a progressive opening (3’ to 5’) of the Hox cluster chromatin is combined with genetic control regions outside the Hox cluster [9,10]. Another proposed mechanistic model is based on the hypothesis that physical forces are responsible for Hox collinearity [11-15]. 

In many animal species Hox genes are clustered in a particular chromosome. The vertebrates possess four paralogous Hox clusters (Hoxa, Hoxb, Hoxc and Hoxd) each one positioned on a different chromosome [2]. On the vertebrate embryo, the genes of a Hox cluster are activated along the anterior-posterior axis of the embryo in a partially overlapping manner (Fig. **1**): the anterior boundary of expression of a Hox gene is shifted posteriorily compared to the anterior boundary of expression of the precedent Hox gene in the sequence Hox1, Hox2, Hox3,…(spatial collinearity). 

The above impressive facts are described in several reviews where the evolutionary context is emphasized [1,2]. The last decade some genetic engineering methods were developed which make possible the accurate intervention in the Hoxd locus and, as a result, transgenic mice are created with deleted or duplicated regions of the Hoxd cluster [9,10]. In other experiments *Hoxb1* is transposed in the *Hoxd* cluster [16]. The produced transgenic expressions are compared to the wild type expressions in the mice limb buds and the trunk [9,10]. This comparison is very interesting since it illuminates several facets of the mechanism responsible for the collinearity of Hox genes. 

Recently Tschopp and Duboule separated the centromeric neighborhood of the Hoxd cluster from the cluster itself, by engineering a large inversion of this centromeric neighborhood. They observe significant differences between the normal and mutant Hoxd expressions during the early embryo stages and they attribute these differences to a regulatory ‘landscape effect’ over the activity of the Hoxd cluster [17]. 

The two mechanistic models mentioned above are suitable to explain the genetic engineering experiments. In the following the two models are described and compared in detail. Furthermore, they are applied in order to reproduce the experimental results. 

## MECHANISTIC COLLINEARITY MODELS 

In order to explain the whole set of data of Hox gene collinearity Duboule and coworkers have proposed the ‘two-phases model’ whose details are found in ref. [9,10,17]. It is a molecular model that functions at the early phase of mouse development (up to about stage E9.5) and the late phase (up to about E12.5). Gene activation is regulated sequentially in time from the telomeric side (3’) of the Hoxd cluster. In the limb a telomeric active site was located, the so-called ELCR (early limb control regulation). This positive activation is balanced by a repressive centromeric influence (POST) [9]. The two influences combine and produce a sequential chromatin opening that leads to a pattern of partially overlapping expressions in the anterior-posterior direction (Fig. **1**). 

**Fig. (2). Mechanical analogue of Hox cluster decodensation and extrusion:**
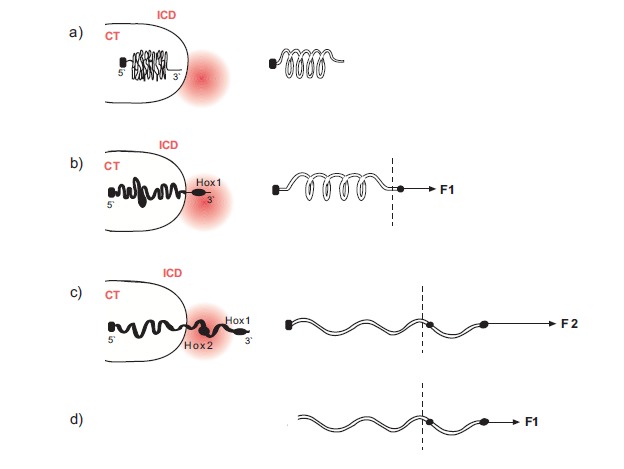
**a**) Before activation the Hox cluster is condensed inside the chromatin territory (CT)-(left). Mechanical analogue: an uncharged elastic spring fixed at its left end (right). **b**) The cluster is slightly decondenced and Hox1 is extruded in the interchromosome domain (ICD) in the area of the transcription factory (TF -red disc) (left). A small force F1 is applied at the loose end and expands slightly the spring (right). **c**) The cluster is further decondenced and the extruded Hox2 is located in the transcription factory area while Hox1 moves away from TF (left). A bigger force F2 > F1 expands further the spring (right). **d**) The posterior fixed end of the spring is cut off. A small force F1 expands and shifts the spring as in c).

An alternative model, coined ‘biophysical model’**,** is presented elsewhere in detail [11-15]. According to this model the macroscopic space and time signals are transduced to the microscopic Hox gene cluster level where forces are generated. A working hypothesis could be the Coulomb forces generated between the negative charges (N) of the gene cluster and the positive charges (P) deposited in its surroundings. These forces decondense and pull the chromatin fiber from inside the chromatin territory (CT) toward the transcription factories (TF) located in the interchromosome domain (ICD) where the genes are activated (Fig. **2**). A mechanical analogue of this mechanism is the elastic expansion of a spring. Cook and coworkers [18] have recently shown that, contrary to common belief, it is the DNA that moves toward the transcription factories where the immobilized polymerases activate the genes. This picture strongly supports the biophysical model hypothesis. When a gene moves away from the ‘factory’ the intensity of its activation drops sharply [19]. This observation offers a natural explanation of quantitative collinearity [15]. The biophysical model successfully describes almost all accumulated findings for the primary anterior-posterior axis and the secondary limb bud axis of vertebrates. The details are found in the publications [11-15]. However, the recent centromeric inversion experiments are not dealt with in these papers. Here, I apply the biophysical model and give a satisfactory explanation of these inversion experiments. Obviously there is a conflict between the biophysical model explanation and the ‘landscape effect’ proposed by Tschopp and Duboule [17]. In the following I propose two experiments which will help to decide which model is correct (if any at all).

## PROS AND CONS FOR THE TWO MODELS

The ‘two-phases model’ is based on well studied mechanisms involving enhancers, inhibitors, promoters and other molecules that regulate the genetic activity. Without excluding these important processes, the ‘biophysical model’ proposes an underlying mechanism that triggers where and when this molecular machinery is activated. Comparing the two models I would like to point out some differences.

**Fig. (3). Hox1 expression as a result of a multiscale action:**
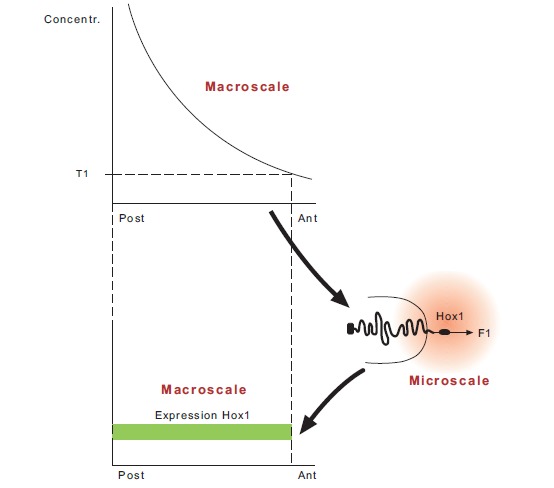
Top: A macroscopic concentration gradient where the threshold T1 determines the domain of space-time signals for Hox1 activation along the anterior-posterior axis. Center (right): The signals are transduced inside the (microscopic) nucleus. A pulling force F1 is generated that activates Hox1. Bottom: Collective gene activation leads to the macroscopic Hox1 expression along the anterior-posterior axis.

1. The two-phases model extends to both early and late developmental phases aiming to explain the observed phenomena during all these stages. In contrast, the validity range of the biophysical model is limited to the early phase only where the mechanism involved is relatively simpler.
2. As already mentioned, Hox gene collinearity is fundamentally a multiscale phenomenon where multicellular (macroscopic) and subcellular dimensions (microscopic) are inherently interconnected. This phenomenon is a characteristic example of systems biology with the multiscale organization requiring, besides the molecular, a multidisciplinary (physical and mathematical) treatment [4].
  The biophysical model establishes such a multiscale interrelation: a spatial and temporal signal in every cell of the multicellular tissue is transduced to the genetic subcellular domain [13]. Subsequently, at the microscopic level, physical forces are created which cause differential Hox gene activation. These microscale forces inherently contain the ‘positional-and-time information’ from the macroscale domain. Subsequently, the genetic activation is collectively transferred to the multicellular level causing the characteristic expression patterns in space and time (Fig. **3**). The transition from the macroscopic to the microscopic and back again to the macroscopic scale is achieved by feedback loops which are indispensable in the multiscale organization of systems biology [4].
  The two-phases model functions at the DNA (microscopic) level. The spatial demarcation of the Hox gene expressions at the tissue level is an observed (macroscopic) result without any causal relation or feedback from the microscopic scale of the model. The phenomena at the two different scales are schematically juxtaposed with no internal connection between them.
3. *Quantitative collinearitry* is naturally explained by the biophysical model: the hox genes approach the transcription factory one after the other and subsequently they move away from it (Fig. **2**).**In this process the closer a gene comes to the polymerase the stronger its expression [14,15]. Quite recent evidence supports the view of chromatin moving toward the immobile polymerase [18,19].
  The two-phases model cannot reproduce the local sequential intensity of hox gene expressions. In order to do this one has to make additional *ad hoc* assumptions.
4. Several experimental findings are unexplained by the two-phases model but well reproduced by the biophysical model. For instance:
  - after the transposition of Hoxb1 in the Hoxd cluster, the cluster decondences but does not loop out [16]. This ‘unexpected’ result is naturally explained by the biophysical model [15].
  - For posterior deletions in the limb bud, according to the two-phases model, ‘unexpected’ redistributions of probe hox genes were observed [9]. These redistributions (posteriorizations) are predicted by the biophysical model [14].
  - For anterior deletions in the trunk, according to the two-phases model, ‘it is impossible to anticipate’ an up-regulation of the mutant hox gene expressions [10]. In contrast the biophysical model predicts an anterior extension of these expressions [14,15].

## A COMBINATION OF EXPERIMENTS SUPPORTS THE BIOPHYSICAL MODEL

In 1999 Kondo and Duboule analyzed two posterior Hoxd deletions [20]:

- **DEL0 **where the posterior [Hoxd13, Hoxd12. Hoxd11] were deleted.
- **DELII** where [Evx2, Hoxd13, Hoxd12. Hoxd11] were deleted.

Following these deletions, the expressions of Hoxd4, and Hoxd10 were examined at the early stages E7.5-E8.5 of mice embryos. It was found that at these stages the **DELII **transgenic Hoxd10 and Hox4 expressions appeared prematurely while the **DEL0** Hoxd10 and Hoxd4 expressions did not appear at all. This was ‘unexpected’ as was also unexpected that at later stages both **DEL0** and **DELII **mutant Hoxd4 and Hoxd10 expressions were comparable to the wild type expressions of Hoxd4 and Hoxd10 respectively.

The biophysical model offers an explanation for these surprising results. These transgenic expressions should be compared to the wild type Hoxd10 and Hoxd4 expressions at the early stages E8-E9. It was found that the wild type Hoxd4 expression at stages E8-E9 is observed posteriorily [10]. This fact can explain the **DEL0** and** DELII **data: the posterior deletions of **DEL0 **lead to a delay of the mutant probe expressions [14,15]. Therefore at E8-E9 the mutant** DEL0** Hoxd4 and Hoxd10 expressions are missing because they have not appeared yet. 

Comparing **DELII **and **DEL0 **we notice the following fundamental difference: in** DEL0 **the posterior deletion does not destroy the elastic spring character of the cluster whereas in** DELII **the fixed posterior end of the cluster is cutoff and the spring is loose at its both ends (Fig. **2d**). As a result, a smaller than normal force acting at the anterior end can decondense and expand the spring [14,15]. The result is a premature expression of the mutant Hoxd4 and Hoxd10 expressions in** DELII** compared to the Hoxd4 and Hoxd10 expressions of** DEL0** in agreement with the Kondo and Duboule results [20]. At later stages, because of secondary regulatory and repairing mechanisms, the mutant expressions become comparable to the wild type expressions. 

## DOUBT FOR THE ‘LANDSCAPE EFFECT’ AT THE EARLY PHASE

**Fig. (4):**
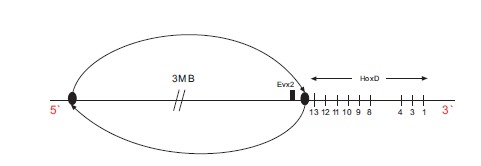
Large inversion centromeric to the HoxD cluster. Evx2 is included in the genetic inversion.

In the recent paper of Tschopp and Duboule the following genetic engineering experiment is described [17]: a large centromeric region neighboring the posterior end of the Hoxd cluster is inverted (Fig. **4**). For this inversion and among other observations, the transgenic expressions of Hoxd13-Hoxd10 at the early stages are compared to the wild type expressions. They find that the transgenic expressions are premature and spatially more extensive. They put forward the hypothesis that in this large centromeric region some smaller regions are contained which produce an inhibitory effect on the Hoxd cluster. Candidate regions causing this inhibitory effect have been localized centromeric to Evx2. Such regions lie e.g. between the Rel3 and Rel2 breakpoints [17, 20]. Following the two-phases model, Tschopp and Duboule [17] conclude that the above inversion relocates the regulatory centromeric region far away from the cluster so that its inhibitory influence on the cluster fades out. As a result, the centromeric ‘landscape effect’ is supressed and the remaining positive telomeric influence causes the observed premature up-regulation of the posterior Hoxd expressions.

The biophysical model proposes a quite different explanation of the above transgenic expressions (Fig. **2**): the mechanical analogue of the Hoxd cluster is the expanding elastic spring with a loose telomeric end (3’) and a fixed centromeric end located between the last gene of the cluster (Hoxd13) and Evx2 [15]. When the fixed end is removed the spring becomes loose at both its ends and it can slide and decondense when a smaller than normal force is applied to the telomeric end (Fig. **2d**). The smaller force is related to a premature and more anterior than normal gene activation at the early stages [14,15]. This argumentation was used to explain the mutant expressions of Hoxd10 and Hoxd9 [15] when the engineered inversion included the posterior subcluster up to Hoxd11 [10]. The same arguments were used above to explain the Kondo and Duboule results [20]. 

The same explanation holds when the inversion starts just after Hoxd13 and it includes Evx2 (Fig. **4**). In this case, the fixed posterior end is cut-off and the elastic spring becomes loose at its both ends. Again, an extended activation (anteriorization) of Hoxd13-Hoxd10 is expected to occur prematurely. This is exactly what Tschopp and Duboule observe (ref. [17]: Fig. **3**). If the biophysical model explanation is correct, the experimental evidence does not necessarily lead to ‘a landscape effect’ for the early stages. On the contrary, it reinforces the picture of the Hox cluster behaving like an elastic spring. At later stages however, secondary and restoring mechanisms get involved and such a ‘landscape effect’ may occur.

## PROPOSAL FOR TWO MODEL-DISTINGUISHING EXPERIMENTS

I presented above two distinct explanations for the experiment where a large centromeric inversion was genetically engineered. Is it possible to conclude which explanation is correct? In the following I propose two experiments which will help to decide.

**Fig. (5):**
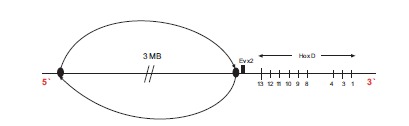
Large inversion centromeric to the HoxD cluster. Evx2 is not included in the genetic inversion.

- Experimental design: in the proposed centromeric inversion shown in Fig. (**5**), the inverted region is almost the same with the reported inversion of Fig. (**4**). The only difference is that in the new inversion the small region between Hoxd13 and Evx2 is unaffected.
  1. Following the two-phases model, this small region cannot significantly influence the ‘landscape effect’ which is due to the much bigger centromeric area that is inverted. Therefore it is expected that, at the early stages, the transgenic expressions of Hoxd13-Hoxd10 for both inversions (Figs. **4** and **5**) should be almost the same: premature and overextended compared to the wild type expressions.
  2. According to the biophysical model, the Hox cluster behaves like an elastic spring whose fixed posterior end lies in the small region between Hoxd13 and Evx2. As long as the fixed end of the spring remains in place the cluster behaves normally. Therefore, for the inversion of Fig. (**5**) during the early stages, the expressions of Hoxd13-Hoxd10 should be very similar to the wild type expressions.
  Summarizing for the centromeric inversion of Fig. (**5**), at the early phase, the two models predict quite different transgenic expressions of Hoxd13-Hoxd10: whereas the two-phases model predicts abnormal expressions (premature and up-regulated) - the biophysical model predicts normal (wild type) expressions. I think it should be of interest if this experiment were ever performed. If the prediction of one of the two models is confirmed, this will lead automatically to the refutation of the other model. There is still the possibility that the experimental result will differ substantially from both model predictions. In this case both models will be in trouble.
- The Kondo and Duboule experiment and its ‘unexpected’ findings [20] indirectly confirm the biophysical model prediction of premature expressions after the deletion of the small region between Evx2 and Hoxd13. Probably it is necessary to perform a deletion experiment where only the small region between Hoxd13 and Evx2 is deleted: according to the two-phases model this small deletion should not affect substantially the Hoxd expressions, so the mutant Hoxd expressions should be similar in space and time to the wild type ones. By contrast, according to the biophysical model arguments presented above, these mutant Hoxd expressions should be prematurely anteriorized in the early phase.

## DISCUSSION

If the findings from the proposed experiments agree with the biophysical model predictions, it should be worth formulating some more detailed models or raising specific questions e.g.: a) what is a realistic distribution of the positive and negative electric charges and, taking into account their relative distances, what is the consequent accurate Coulomb force F? b) what is the degree of reversibility of the chromatin expansion -how elastic is the ‘spring’? c) besides the Coulomb forces are there some other forces that can pull the chromatin fiber? d) If the ‘landscape effect’ is confirmed as a late effect, at what stage does it come into play and where are its origins located? 

The Evx2-Hoxd13 intergenic region of 8 kb was examined in detail and it was found that it plays a role as a boundary element with a differential spatial and temporal activity [21]. From this analysis it is clear that the deletion of this intergenic region is not expected to cause an effect on the Hoxd cluster comparable to the biophysical model prediction –premature and anteriorily ectopic Hoxd expressions in the early stages. 

The understanding in evolutionary terms remains questionable of how or why a disordered long cluster is consolidated into an ordered shorter cluster [2]. The biophysical model might hint toward a sensible solution. The negative charges of a disordered long cluster are dispersed and they cannot generate a unidirectional electric force. In contrast, a condensed short cluster is selectively favorable since the resultant electric force is directionally more efficient in pulling the cluster from its 3’ end. 

The genetic engineering experiments analyzed here refer to interventions in the DNA and the negative charge (N) of the Hoxd cluster. However, some other experiments are reported which indirectly deal with manipulations of the positive charge (P) of the chromatin environment. Such an experiment was performed by Vargesson *et al. *[22]. In this experiment beads soaked in FGF4 were implanted at the distal posterior tip of a chick limb bud. This implantation produces an excess of the signal transduced to the Hoxa cluster environment and, according to the biophysical model, the increase of P can lead to a stronger pulling force F that extrudes Hoxa13 further away from the TF area (see Fig. **2c**). This gene shift could explain why the Hoxa13 expression fades out around the implanted bead area [22]. Furthermore, this picture ties up with the hypothesis that a Hox gene can be expressed in a region where the appropriate morphogen concentration lies between a lower and an upper threshold [22,13]. This concentration range in the macroscopic scale could then be associated to the microscopic activation region around the transcription factory of Fig. (**2**). Note that this association is another manifestation of the multiscale entanglement that causes Hox gene collinearity.

After the submission of the present article, a paper appeared which deals with the three-dimensional architectural changes of the HoxD cluster *in vivo* before and after Hox transcriptional activation in mouse embryos [23]. This report strongly supports the biophysical model since it confirms that during Hox activation the genes are progressively translocated in space from an inactive domain to a transcriptionally active compartment [23]. This is exactly a basic biophysical model prediction. In contrast, this translocation is not expected according to the two-phases model.
